# Determining the Composition of Lignins in Different Tissues of Silver Birch

**DOI:** 10.3390/plants4020183

**Published:** 2015-04-09

**Authors:** Kurt V. Fagerstedt, Pekka Saranpää, Tarja Tapanila, Juha Immanen, Juan Antonio Alonso Serra, Kaisa Nieminen

**Affiliations:** 1 Department of Biosciences, Division of Plant Biology, P.O. Box 65, University of Helsinki, FI-00014 Helsinki, Finland; E-Mails: juha.immanen@helsinki.fi (J.I.); juan.alonsoserra@helsinki.fi (J.A.A.S.); 2 Natural Resources Institute Finland, Jokiniemenkuja 1, Fi-01300 Vantaa, Finland; E-Mails: pekka.saranpää@luke.fi (P.S.); tarja.tapanila@luke.fi (T.T.); kaisa.nieminen@luke.fi (K.N.); 3 Institute of Biotechnology, P.O. Box 65, Helsinki University, FI-00014 Helsinki, Finland

**Keywords:** acetyl bromide, *Betula pendula*, cupric oxide, lignin analysis methods, phloem, thioacidolysis, xylem

## Abstract

Quantitative and qualitative lignin analyses were carried out on material from the trunks of silver birch (*Betula pendula* Roth) trees. Two types of material were analyzed. First, whole birch trunk pieces were cryosectioned into cork cambium, non-conductive phloem, the cambial zone (conductive phloem, cambium and differentiating xylem), lignified xylem and the previous year’s xylem; material that would show differences in lignin amount and quality. Second, clonal material from one natural birch population was analyzed to show variations between individuals and between the lignin analysis methods. The different tissues showed marked differences in lignin amount and the syringyl:guaiacyl (S/G) ratio. In the non-conductive phloem tissue containing sclereids, the S/G ratio was very low, and typical for phloem fibers and in the newly-formed xylem, as well as in the previous year’s xylem, the ratio lay between five and seven, typical for broadleaf tree xylem. Clonal material consisting of 88 stems was used to calculate the S/G ratios from the thioacidolysis and CuO methods, which correlated positively with an R^2^ value of 0.43. Comparisons of the methods indicate clearly that the CuO method is a good alternative to study the monomeric composition and S/G ratio of wood lignins.

## 1. Introduction

Lignin is an aromatic, heterogenic cell wall heteropolymer that is essential for mechanical support, water transport and disease resistance in trees [1]. Lignin can be fully removed, but only under drastic chemical conditions using either strong acid (e.g., sulfite pulping process) conditions under elevated temperatures and pressures or with ionic solvents. As the removal of lignin is costly, there has been considerable interest in reducing lignin content by genetic engineering of trees as a means of improving the efficiency of the pulping process, reducing the use of chemicals and improving profitability [2]. Lignin renders wood xylem recalcitrant in the biorefinery processes when bioethanol is being produced. Lignin is also a valuable polymer and serves as a platform chemical for various compounds, replacing fossil raw materials, such as carbon fibers, dispersants, vanillin and new types of plastics [3]. Hence, it is important to know the lignin composition to understand how it can be processed further into new products.

Deciduous wood lignin is composed of syringyl (S) and/or guaiacyl (G) units linked by a series of ether and carbon-carbon bonds. The ether β-*O*-4 linkages are both frequent and labile, which makes them the target of the delignification process. In contrast, the carbon-carbon linkages are resistant, especially the biphenyl 5–5 bonds involving the aromatic C-5 position, which is available for interunit linkages only in G units. Thus, conifer wood lignin essentially made of G units is less susceptible to kraft delignification than deciduous wood lignin [4].

The most commonly-used methods for quantitative analysis of lignin are the Klason and AcBr methods. Klason is a gravimetric method and only measures insoluble material after hydrolysis with 72% H_2_SO_4_. It is often combined with spectrophotometric determination of dissolved lignin [5]. Klason is regarded as being generally reliable for woody stem material. Recently, this procedure has been modified to allow application to a sample size of 50 mg [6]. The accuracy of lignin determination depends also on tissue and cell wall type [7]. Various non-lignin components, such as tannins, non-extracted polysaccharides and proteins [7,8], can be present in the lignin residue and, thus, limit the applicability of the Klason method in tissues other than xylem. The acetyl bromide method has been reported to be the simplest and fastest among the methods evaluated, presenting similar or best recovery of lignin in all tissues assessed [9]. It is based on the solubilization of lignin and the determination of absorbance values at 280 nm. The acetyl bromide protocol is based on the formation of acetyl derivatives in non-substituted OH groups and bromide replacement of the Cα-OH groups to produce a complete solubilization of the cell wall material under acidic conditions. However, an overestimation of the lignin content can occur due to the oxidative degradation of structural polysaccharides (e.g., xylans) during the incubation of the cell wall with the acid solution [9,10]. One difficulty with the acetyl bromide method is the need for a well-defined lignin standard to calibrate the method [7].

Chemical degradation methods are worthwhile when compositional information on lignin is needed. Thioacidolysis has been applied to a wide variety of materials and isolated lignins [11,12]. Thioacidolysis is an acid-catalyzed method that results in β-*O*-4 cleavage. The detection of C_6_C_3_ products (*p*-hydroxyphenyl (H), guaiacyl (G) and syringyl (S) monomers) gives information on the composition of non-condensed alkyl aryl structures, and the results can be used for calculating the S/G-ratios.

Alkaline cupric (II) oxide oxidation (CuO) is a technique commonly used to analyze the composition of lignins in complex sample matrixes, such as soils and sediments [13,14]. Relatively mild oxidation with CuO induces cleavage of β-*O*-4 ether bonds in lignin. The lignin macromolecule is hydrolyzed, yielding phenolic CuO oxidation products with aldehydic, ketonic and acidic side chains [15,16,17]. The degradation products obtained by both thioacidolysis and CuO methods can be analyzed by a variety of means, including gas chromatography/mass spectrometry (GC/MS). Neither thioacidolysis nor the CuO method completely depolymerize lignin, and therefore, the result does not cover the total content of lignin, but gives valuable information about the lignin substructures.

The aim of the present piece of work is to study the compositions of birch lignins using both the CuO and thioacidolysis methods. For this purpose, we chose the wood material carefully: First, to use the methods on wood material of natural variability, we used 22 different birch clones created to represent different genotypes found in natural stands. Second, to estimate how large variations in the sample ranging from outer phloem to mature xylem will affect lignin composition, we used longitudinal cryosections carefully cut from all of the different tissues of the silver birch trunk. Hence, we had material that contained different types of lignin (phloem fibers *vs.* secondary xylem), high amounts of pectin, proteins and hemicelluloses (cambial region) and tissues with high concentrations of hardwood-type lignin with a high S/G-ratio. Our major aim was to test the reliability and pertinence of the CuO and thioacidolysis methods.

## 2. Results and Discussion

### 2.1. Lignin Composition of Different Silver Birch Tissue Fractions

**Figure 1:**
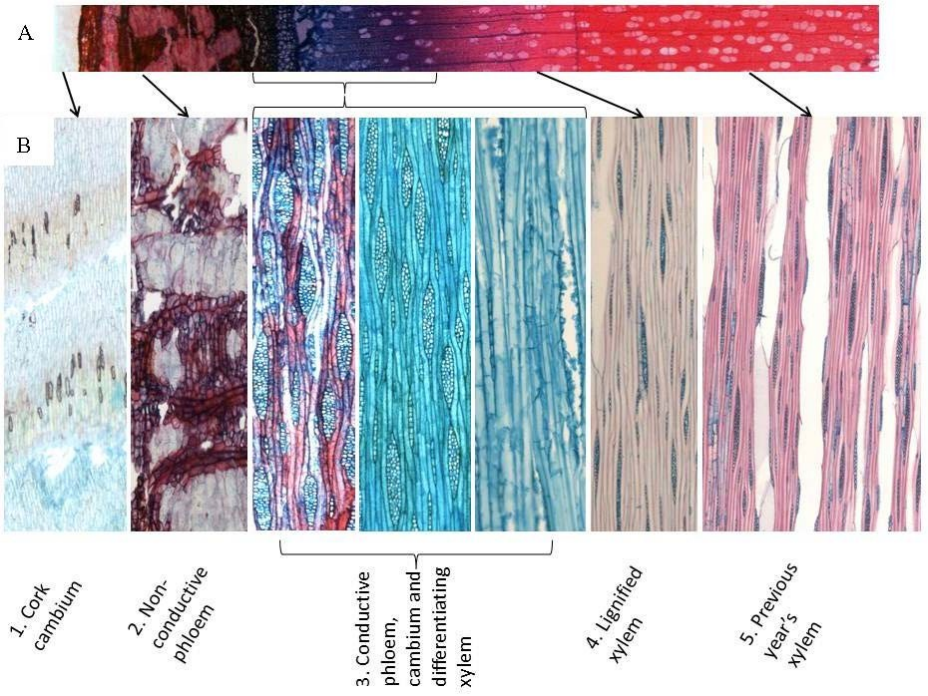
Photomicrographs of the silver birch sample sections with a cryomicrotome. (**A**) A cross-section through the stem. Mainly cellulosic cell walls are stained blue with Alcian blue and lignified tissues red with safranin. (**B**) Longitudinal sections presenting the actual samples for lignin analyses: 1, cork cambium; 2, non-conductive phloem; 3, combined conductive phloem, cambium and differentiating xylem; 4, lignified xylem; 5, previous year’s xylem.

**Figure 2:**
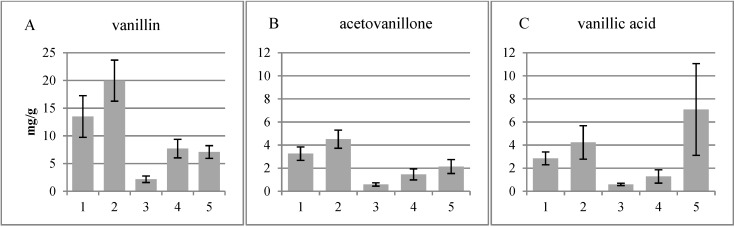

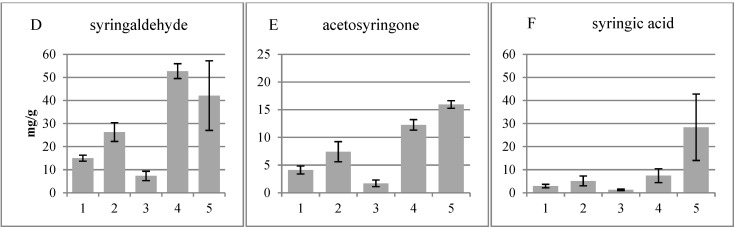
Guaiacyl (**A**–**C**) and syringyl (**D**–**F**) units released by the CuO treatment of the different tissues analyzed with the GC-MS. 1, Cork cambium; 2, non-conductive phloem; 3, combined conductive phloem, cambium and differentiating xylem; 4, lignified xylem; 5, previous year’s xylem.

**Figure 3:**
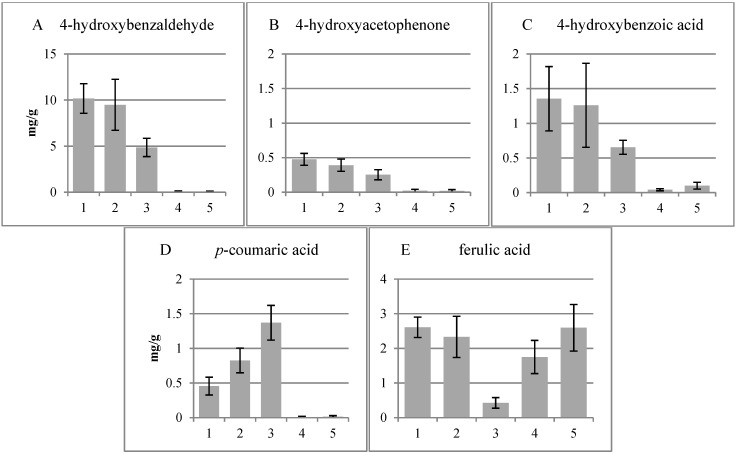
*p*-Hydroxyl phenols (**A**–**C**) and hydroxycinnamyls (**D**,**E**) released by the CuO treatment. 1, Cork cambium; 2, non-conductive phloem; 3, combined conductive phloem, cambium and differentiating xylem; 4, lignified xylem; 5, previous year’s xylem.

**Figure 4:**
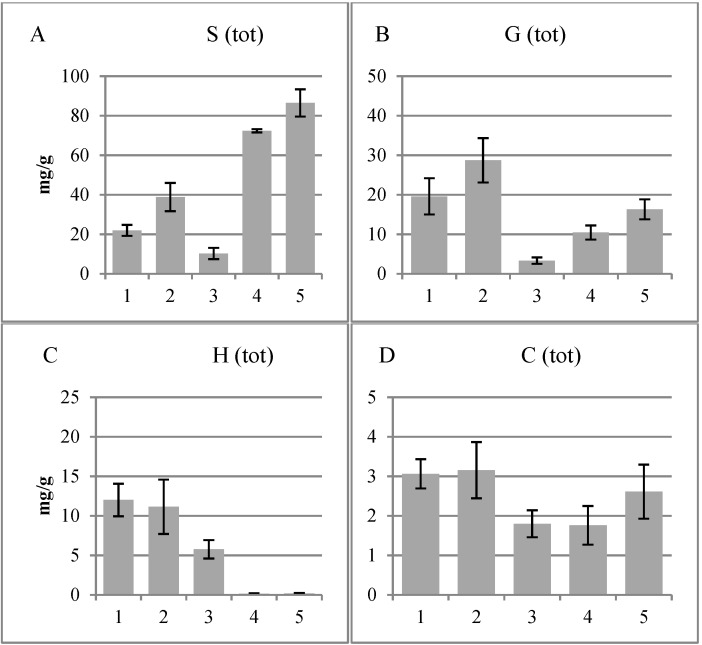
Total amounts per g of dry weight of the syringyl (S)-, the guaiacyl (G)-, *p*-hydroxyphenyls (H)- and hydroxycinnamyls (C)-groups in the different fractions obtained using the CuO method. (**A**) The S-group contains syringaldehyde, acetosyringone and syringic acid; (**B**) the G-group vanillin, acetovanillone and vanillic acid; (**C**) the P-group 4-hydroxybenzaldehyde, 4-hydroxyacetophenone and 4-hydroxybenzoic acid; and (**D**) the C-group *p*-coumaric acid and ferulic acid. 1, Cork cambium; 2, non-conductive phloem; 3, combined conductive phloem, cambium and differentiating xylem; 4, lignified xylem; 5, previous year’s xylem.

Different tissues were cryosectioned from 13-year-old whole silver birch stems and divided into five fractions (Figure 1). The fractions were subjected to CuO treatment to cleave β-*O*-4 ether bonds in lignin. The phenolic compounds derived are shown in Figure 2, Figure 3, and Figure 4. The four major groups include guaiacyl (G), syringyl (S), hydroxycinnamyl (C) and *p*-hydroxyphenyl (H) moieties.

The fractions showed distinctive differences in the products of CuO treatment (Figure 2; note the different scales in the graphs). Aldehydes (vanillin and syringaldehyde) were the major products, with most of the vanillin in the non-conductive phloem, while syringaldehyde was the major product in the lignified xylem. The acid content (vanillic and syringic acid) increased from cork cambium to the xylem. Large variations between the samples were noticeable in the acids.

4-Hydroxy compounds were the products detected in Fractions 1 to 3, but there were only traces in the xylem. Within the hydroxycinnamyl group, ferulic acid was the dominating compound with minor amounts in the xylem (Figure 3). The amount of *p*-coumaric increased from the cork cambium to the cambium and was detected only in minor amounts in the xylem. The amount of ferulic acids in all fractions was at about the same level, except in the cambial zone (Figure 3). Ferulic acid has been shown to take part in the lignin polymer, and it has been postulated as an initiation or nucleation site at the beginning of lignin polymerization [18].

**Figure 5:**
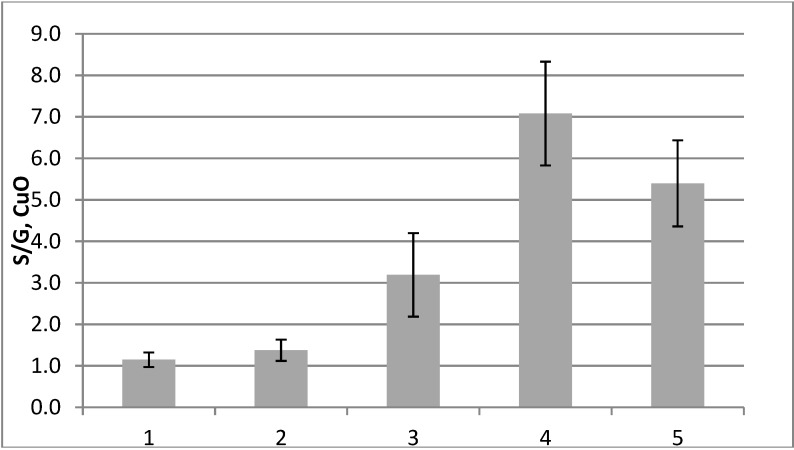
The S/G ratios as calculated from the CuO analysis results. 1, Cork cambium; 2, non-conductive phloem; 3, combined conductive phloem, cambium and differentiating xylem; 4, lignified xylem; 5, previous year’s xylem.

Figure 4 and Figure 5 show the amounts per g of dry weight of the S-, G-, P- and C-groups and the S/G ratios in the different fractions. The S-group contains syringaldehyde, acetosyringone and syringic acid, the G-group vanillin, acetovanillone and vanillic acid, the P-group 4-hydroxybenzaldehyde, 4-hydroxyacetophenone and 4-hydroxybenzoic acid and the C-group *p*-coumaric acid and ferulic acid. The S/G ratio was low in the cork cambium and non-conductive phloem, intermediate in the cambial region and high in the xylem. In the cambial region, the S- and G-group compounds were at a low level.

**Figure 6:**
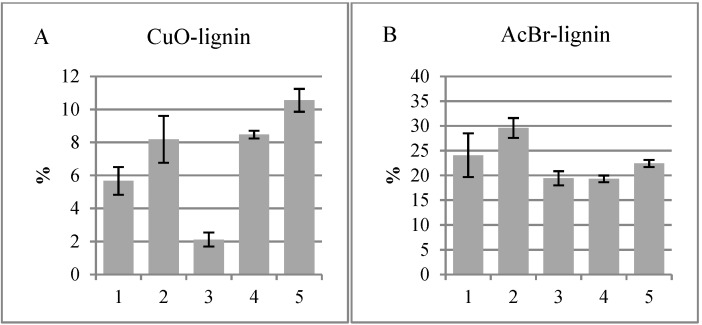
The same silver birch tissues analyzed by CuO and AcBr methods. (**A**) The sum of the monomers released by the CuO method; (**B**) the total lignin amount estimated by the AcBr method. 1, Cork cambium; 2, non-conductive phloem; 3, combined conductive phloem, cambium and differentiating xylem; 4, lignified xylem; 5, previous year’s xylem.

Figure 6B shows that the total lignin contents, determined using the acetyl bromide method, of the different fractions were similar. This is in accordance with the sums of the monomer concentrations released by the CuO method (Figure 6A) for all of the other fractions, except Fraction 3. This fraction, which contained the cambial region where lignification is likely to be at the minimum, yielded the lowest sum of monomers. The absence of lignin in this tissue can also be seen in the staining in Figure 1. This region contains predominantly cellulose, hemicelluloses, pectin and proteins [19]. This discrepancy between Figure 6A,B could be partially explained by the fact that the *p*-hydroxycinnamic acids are bifunctional molecules with carboxylic and phenolic binding sites, and they can be involved in both ester and ether linkages to other cell wall components. It is also true that degraded xylans (due to perchloric acid) have strong absorbance at 280 nm, leading to overestimation of lignin with the AcBr method. The cambial region is high in protein content [19], but proteins precipitate during sample preparation and should not interfere with the AcBr method.

### 2.2. Variation in Lignins in a Natural Silver Birch Population

**Figure 7:**
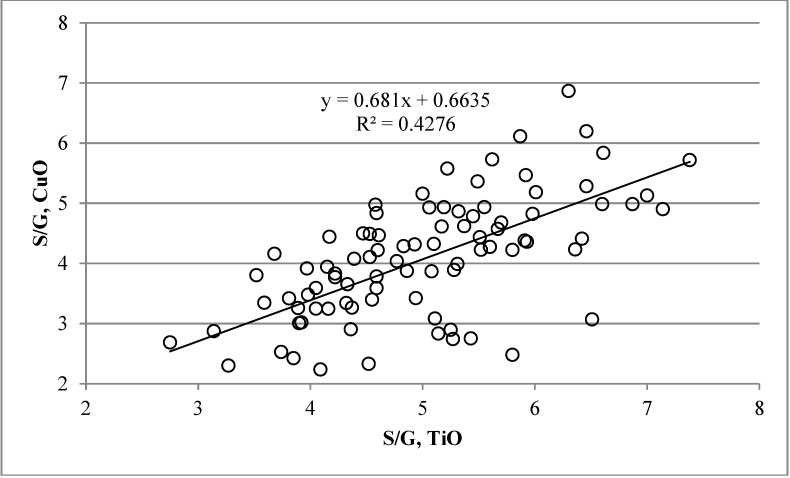
Comparisons of the S/G ratios obtained from the CuO (S/G, CuO) and thioacidolysis (S/G, TiO) analysis results. The wood material originated altogether from 88 trees of 22 different genotypes presenting natural variation in silver birch.

Figure 7 shows a comparison of CuO and thioacidolysis in the estimation of the S/G ratio. The positive correlation was rather low with R^2^ being 0.43 and the linear regression equation y = 0.681x + 0.6635. It was observed that the S/G values obtained with the thioacidolysis method were in most cases higher than those calculated from CuO results (average CuO = *ca.* 80% of thioacidolysis). The interesting and large variation in the S/G ratio in the silver birch xylem material is explained by the fact that the material presented the large natural variation within a natural birch population. It would be of interest to see whether this is reflected in the pulping properties of the wood material. However, the total lignin content (Klason + acid-soluble lignin) showed very little variation, and most samples fell from 23% to 25% of dry weight. The sum of the analytical monomers released by the CuO method reached 25% to 55% of the Klason lignin. In earlier accounts, thioacidolysis covers *ca.* 20%–40% of the weight of the lignin presumably present [20].

## 3. Experimental Section

For the different tissues of the silver birch trunk (*Betula pendula* Roth), we felled three birch trees at the experimental field of the Viikki Campus at the University of Helsinki. The trees were of the clone No. V5834 created from material of the Punkaharju experimental site of the Finnish Forest Research Institute. The trees were 13 years old, *ca*. 10 m in height and their diameter *ca*. 11 cm at a 1.5-m height. Longitudinal sections (20 μm in thickness) were cut in the tangential direction in whole birch trunk pieces with a cryomicrotome and divided into five fractions—1, cork cambium; 2, non-conductive phloem; 3, conductive phloem, cambium and differentiating xylem; 4, lignified xylem; 5, previous year’s xylem—to show differences in lignin amounts and quality between the tissues. For microscopy, the sections were stained with 1% safranin and then 1% Alcian blue, which stain lignified cell walls red and unlignified cell walls blue, respectively.

A field experiment in Punkaharju, southeastern Finland (61°48' N, 29°18' E), was established in 1999 for long-term monitoring of within-stand differences between silver birch (*Betula pendula* Roth) genotypes in growth phenomena. The experiment was planted on abandoned former agricultural land with a fine sandy till soil and had a randomized complete block design with 6 replicate blocks. All blocks contained four replicate trees of the 22 genotypes at a planting distance of 2 × 2 m. The genotypes were micro-propagated from trees selected randomly in one stand (one hectare in size) of *B. pendula* that was naturally regenerated after logging operations in 1979. In 2008, one tree per each genotype in four randomly selected blocks was felled, altogether 88 trees. Sample discs were cut at breast height (1.3 m). Three–four of the outermost annual rings were removed from two cardinal directions (south and north) in order to avoid any fungal infection and decay near the pith. The samples were further cut into 2 × 2-mm sticks.

### 3.1. Quantitative Analysis of Lignin

#### 3.1.1. Klason and Acid-Soluble Lignin Determination

Samples were ground frozen with a blade-mill (Polymix PX-A10). The dry solids content of the milled wood samples was determined at 103 °C. The samples of air-dried wood powders (3 g) were extracted with acetone using a Soxhlet apparatus for 6 h [21]. After evaporation of the solvents, the residues were dried at 103 °C, allowed to cool in a desiccator and then weighed. The amount of acid-insoluble lignin was determined by the Klason method [5]. The samples of the extracted wood powders (300 mg) were treated with 3 cm^3^ of 72% sulfuric acid under vacuum for 1 h. The mixtures were diluted with about 82-cm^3^ portions of water and autoclaved at 125 °C for 1 h. The precipitates were collected with glass fiber filter SS GF 52 × 47 mm by suction filtration and washed with water. The filters with the acid-insoluble lignin (Klason lignin) were dried at 103 °C, cooled in the desiccator and weighed. In order to determine the amount of acid-soluble lignin, the filtrates were diluted with water to 250 cm^3^. Absorption of the acid solutions with the dissolved lignin was measured at 203 nm using sulfuric acid of the same concentration as a blank.

The absorbance readings were obtained with a Shimadzu UV-2401 PC UV-VIS Recording spectrophotometer. The total lignin (Klason lignin + acid-soluble lignin) content was calculated from the unextracted wood as follows: Klason lignin% = p(100 − u)/m, in which p = precipitate (g), u = extractives (%) and m = calculated dry weight of extracted sample (g). The acid-soluble lignin content was calculated using a lignin absorptivity of 128 L·g^−1^·cm^−1^ and corrected because of the absorption of carbohydrates according to [21]. The total lignin content of each sample was determined as the mean of the duplicate measurements; this is referred to as the measured lignin content of the sample.

#### 3.1.2. AcBr-Based Determination

The powder was either lyophilized (birch tissues) or dried at 60 °C for 48 h (birch clones), and 5- or 10-mg samples were extracted with acetone to remove soluble extractives. Lignin contents were determined using the acetyl bromide method [22,23]. A sample of 5 or 10 mg of phloem and inner bark tissue was weighed into a 10-mL screw-capped test tube and sonicated with 5 mL of acetone for 30 min. The extract was pipetted off, and the extractive free sample was dried. It was redissolved in 5 mL 20% (v/v) AcBr-acetic acid solution (containing 100 µL 70% perchloric acid, a hazardous chemical) and the sample was kept in a block heater at 50 °C for 3 h with regular shaking [22]. After treatment, the sample was frozen at −20 °C for 15 min in order to stop the reaction. The melt solution was transferred to a 50-mL volumetric flask containing 5 mL 2 M NaOH and 12 mL 100% acetic acid. The solution was diluted into 50 mL with acetic acid. The UV spectrum was measured with a Shimadzu UV-2401 spectrometer at 280 nm. Lignin content was calculated using the following expression:

Lignin% = 100(As − Ab)V/aW

As = absorbance of sample
Ab = absorbance of blank
V = volume of solution
W = weight of sample
a = the absorptivity of a lignin standard calculated for each analysis series

AcBr determination was calibrated with the total lignin content (Klason + acid-soluble) of a silver birch wood sample (lignin standard).

### 3.2. Qualitative Analysis of Lignin

#### 3.2.1. Thioacidolysis: Method, TiO

The syringyl:guaiacyl (S/G) determinations of the wood lignin were made by the modified method of thioacidolysis [12]. An extractive-free wood sample (10–30 mg) was mixed with 3 mL of freshly prepared thioacidolysis reagent (2 M BF_3_ etherate in an 8.75:1(v/v) dioxane/ethanethiol mixture). The thioacidolysis proceeded at 103 °C for 4 h. After cooling, the reaction mixtures were rinsed with water in the reaction tube containing methylene chloride (CH_2_Cl_2_). The lignin fragments were extracted from the aqueous phase thrice with 2 mL CH_2_Cl_2_. The organic fractions were combined, dried by the addition of anhydrous Na_2_SO_4_ and evaporated under nitrogen. Before the GC-MS analysis, the dried samples were silylated with 0.5 mL 20% TMSI-pyridine mixture (TMSI = 1 − (trimethylsilyl)imidazole) at 60 °C for 1 h, and then, the silylation was continued at room temperature overnight. The GC-MS analyses were performed using a HP 6890 GC-system equipped with a Mass Selective Detector 5873 and an HP-5 capillary column (30 m × 0.25 mm i.d., 0.25 μm film thickness). Helium was used as a carrier gas, with a flow of 1.5 mL/min.

The mass spectral characterizations were done according to Rolando *et al.* [23]. S/G ratios were defined as peak area ratios of syringyl and guaiacyl monomeric erythro/threo isomers, assuming that they have similar response factors, which is the case [11].

#### 3.2.2. Alkaline Cupric (II) Oxidation: Method, CuO

Alkaline cupric (II) oxidations were carried out according to the procedure developed by Hedges and Ertel [16]. Some modifications, including those introduced by Goni and Montgomery [24], were made. Oxidations were performed using a microwave digestion system MSD-2000 and a liquid phase hydrolysis accessory set (CEM Corporation). A known amount of sample, typically 50 mg, 15 mL 2 M NaOH (N_2_-spurged), 500 mg CuO, 50 mg Fe(NH_4_)_2_(SO_4_)_2_•6H_2_O and 50 µL of internal standard solution (2 mg/mL of cinnamic acid) were added into the reaction vessel. The reaction vessel (120 mL) covers were sealed, and the closed reaction vessels were connected to each other via the Teflon tubing and loaded into the microwave oven. The fiber-optic temperature probe (Thermo-Optic) was connected to the first reaction vessel and the pressure/vacuum line to the last reaction vessel. Air was removed from the reaction vessels and was replaced by N_2_-gas. The microwave-assisted CuO-treatment of samples was done at 150 °C for 1.5 h in a N_2_ atmosphere.

After oxidation, the cooled solutions were acidified, and the CuO reaction products were extracted with 4 mL of ethyl acetate three times. The water present in the combined ethyl acetate extract was removed by the addition of Na_2_SO_4_. After evaporation, the extracts were silylated with N,*O*-bis(trimethyl-silyl)trifluoroacetamide (BSTFA) with 1% trimethylchlorosilane (TMCS). The GC-MS detection of trimethylsilyl derivatives of CuO oxidation products was done with the same GC-MS instrument as was used in the thioacidolysis method.

The phenolic compounds derived by CuO oxidation were divided into four different structural groups; guaiacyls (G), syringyls (S), *p*-hydroxyphenyls (H) and hydroxycinnamyls (C). The vanillyl, syringyl and *p*-hydroxyl groups consist of corresponding aldehyde, ketone and carboxylic acid. The hydroxycinnamyl group includes ferulic acid and *p*-coumaric acid. Quantification of individual compounds was performed using the relative response factors adjusted to the peak area of internal standard and individual lignin phenols. The response factors were calculated from the analysis results of standard solutions. The amounts of different structural groups, as well as the total lignin content were counted as the sum of individual compounds.

## 4. Conclusions

Lignin is a complex macromolecule, and hence, it has been stated many times that there is no single method for the accurate determination of the quality or quantity of lignin [8,25]. Because of the high molecular weight and insolubility, knowledge of the composition of wood lignin is obtained indirectly, e.g., by the identification of lignin degradation products. This can be done with CuO and thioacidolysis methods, which give valuable information on the monolignol composition of lignin. Oxidation with CuO induces cleavage of β-*O*-4 ether bonds in lignin and retains the three carbons of the side chain of the phenol, and the lignin macromolecule is hydrolyzed.

As we have shown, CuO and thioacidolysis give different degradation products and, depending on how they are included in the calculations, give different S/G ratios. Another possible reason is that in the CuO method, monomers are also released from other units, such as β-5. The reason for the differences may be because the CuO method is perhaps also capable of releasing monomers from other units, such as ß-5. In addition, direct comparisons of S/G-ratio lignin data generated from the CuO and thioacidolysis methods are difficult, as different lignin subunits were analyzed in each process; however, the components are analogous. Hence, it is vital that for comparisons, the S/G-ratios should be the results of the same analytical method.

Downscaling of the sample size presents another problem: representative analysis and results are challenging if the sample size is on a mg scale as, e.g., vessels and fibers behave differently in grinding and may result in uneven samples. This is more accentuated in samples containing other tissues than xylem as, e.g., phloem fibers differ from xylem in the S/G ratio. Both the CuO and thioacidolysis results show clearly that silver birch phloem sclereids have a much lower S/G ratio than xylem. Similar results have been found in poplar tissues by thioacidolysis [19].

In summary, we can say that the different silver birch tissues showed marked differences in lignin amount and S/G ratio, and the results differed among the methods tested. The S/G ratios calculated from the thioacidolysis and CuO results correlated positively, but with an R^2^ value of 0.43. This means that the S/G-ratios, as well as other qualitative lignin parameters should always be determined with the same method if comparisons between tissues and species are made. However, this work indicates clearly that the CuO method, which has been used earlier mainly for soil analyses, is a good alternative to study the monomeric composition and S/G ratio of wood lignins without the drawbacks of thioacidolysis.
